# Is the Natural Instinct to Oviposit in Mated Female Oriental Fruit Fly, *Bactrocera dorsalis* More of a Brain-Independent Act?

**DOI:** 10.3389/fphys.2022.800441

**Published:** 2022-03-14

**Authors:** Meenal Vyas, Saravan Kumar Parepally, Pagadala Damodaram Kamala Jayanthi

**Affiliations:** Division of Crop Protection, ICAR-Indian Institute of Horticultural Research, Bengaluru, India

**Keywords:** tephritids, egg-laying, decapitation, differential gene expression, γ-octalactone, oviposition stimulant, oviposition site selection

## Abstract

What physiological and neuro-molecular changes control the female oviposition behavior post-mating in insects? The molecular changes that occur in a gravid female insect are difficult to dissect out considering the distinct behavioral patterns displayed by different insect groups. To understand the role of the brain center in Oriental fruit fly, *Bactrocera dorsalis* oviposition, egg-laying behavior was analyzed in γ-octalactone exposed, decapitated mated *B. dorsalis* females. Interestingly, the females displayed a possible urge to oviposit, which suggests a natural instinct to pass on the gene pool. Expression analysis of certain genes involved in oviposition behavior was also carried out in these insects to explore the molecular aspects of such behavior. This study tries to assess the involvement of brain center in egg-laying and also explore the role of certain neurotransmitter-related receptors in decapitated *B. dorsalis* oviposition behavior. Our results indicate that *B. dorsalis* oviposition behavior could potentially have a bypass route of neuronal control devoid of the brain. The study reported here establishes that decapitation in gravid females fails to abolish their ability to sense ovipositional cues and also to oviposit.

## Introduction

The fate of the insect world rests on insect mothers. As a community, insects have often been regarded successful in the evolutionary sense because of their extremely high fecundity and resilience. The switch from a mating-oriented behavior to an oviposition site-seeking behavior is almost immediate in most insects, and the associated molecular switches have also been documented in some tephritids (Jang, 1995; Gillott, 2003; Gomulski et al., 2012; Córdova-García et al., 2021; Devescovi et al., 2021) and *Drosophila* (McGraw et al., 2008). Oviposition in insects is driven by semiochemical cues that elicit a response from the gravid females that triggers the downstream reactions such as when and where to lay eggs (reviewed in Kamala Jayanthi et al., 2014a,b, 2017, 2021; Cury et al., 2019), finally resulting in egg-laying. Several factors drive oviposition in insects: availability of food (Stahlschmidt et al., 2014), assessment of threat from conspecifics (Shelly, 1999; Ekesi et al., 2009), natural predators (Van Mele et al., 2009), and most importantly, ovipositional stimulation and cues from the host (Freeman and Carey, 1990; Kamala Jayanthi et al., 2012, 2014a,b). Insects carry the memory of ovipositional stimulant cues that are a part of the host volatiles which support their progeny survival (Gregório et al., 2012; Kamala Jayanthi et al., 2014a). A gravid female insect chooses the best possible oviposition sites to provide an environment suitable for the proper development of its progeny (Fontellas-Brandalha and Zucoloto, 2004; Rattanapun et al., 2009). In higher-order organisms such as humans, decision-making is driven by strong emotions. For insects, it is a constant learning process and their decisions are guided by various cues that include environmental stimuli. Ovipositional stimulus is guided by different coordinating tissues, such as the endocrine system, the female reproductive tissues, and neuronal network aside from the molecular effectors that are involved in the entire process (reviewed in Cury et al., 2019). Pathways with molecular elicitors and neurotransmitters that influence oviposition have been identified, and the octopamine–tyramine pathway (reviewed in White et al., 2021) is one among them. Octopamine and its receptors are involved in oviposition in *Plutella* (Li F. et al., 2020), whereas mutants of the octopamine tyramine receptors caused reproductive sterility in *Drosophila* (Lee et al., 2003; Lim et al., 2014; Li et al., 2015). Proctolin, a contracting muscle protein involved in the ovipositor muscle contractions (Noronha and Lange, 1997) has a suggested role in insect oviposition. Vitellogenin and a few olfactory genes are also involved in oviposition (reviewed by Li H. et al., 2020).

As the whole insect body reacts to the post-mating switch, what role does the insect brain have in oviposition? Studies done in *Drosophila* suggest that there are specific olfactory neurons and receptors that trigger oviposition (Chin et al., 2018). Therefore, some sites in the brain center control oviposition. Decades ago, studies done on decapitated *Drosophila* females suggested the presence of alternate mechanisms or neural networks that guide oviposition. Among the examined Drosophilids, *Drosophila melanogaster*, *Drosophila pseudobscura*, and *Drosophila tripunctata* were capable of egg-laying post-decapitation while *Drosophila virilis* and *Drosophila palustris* failed to do so (Grossfield and Sakri, 1972). In the Jamaican biting midge, decapitation is known to induce oviposition because of the loss in inhibition associated with the brain neural network (Linley, 1965). Similar results have been noticed in grasshoppers (Thompson, 1986), silkworms (M’Cracken, 1907), western corn rootworm (Spencer and Orellana, 2020), and crane flies (Chiang and Kim, 1962). Whereas most of the studies that are related to oviposition and decapitation suggest reflexive action, we hypothesize that it could be a maternal urge to pass on the gene pool to next generation. Post-oviposition maternal care is evident in many insects that include Hemipterans and Heteropterans (Tallamy and Schaefer, 1997; Gogala et al., 1998; Guilbert, 2003), but no studies explore the aspect of a selfish urge to contribute to the next generation. Does the brain center guide oviposition in *Bactrocera dorsalis* or does egg-laying occur independently of the brain under stressful conditions? To answer some of these questions, egg-laying behavior was investigated in decapitated female *B. dorsalis*, an economically important polyphagous pest of several horticultural crops. Using *B. dorsalis* as the model system, our research group is trying to understand the chemoecological behavior with respect to host attraction and oviposition (Kamala Jayanthi et al., 2012, 2014a,b, 2017, 2021). Chemical ecology studies done on oviposition response of *B. dorsalis* to stimulants from host plant volatiles have helped identify a robust ovipositional stimulant, γ-octalactone (GOL hereafter) (Kamala Jayanthi et al., 2014a). In this study, GOL was used as an ovipositional stimulant, and oviposition under the stimulation of GOL was observed. The expression profile of some predicted molecular elicitors of oviposition behavior was determined in decapitated and intact females exposed to GOL. Results obtained support our hypothesis that the brain center has limited control over oviposition in *B. dorsalis* females.

## Materials and Methods

### Insect Rearing

*Bactrocera dorsalis* (Hendel) (Diptera: Tephritidae) flies (locally collected) were reared and maintained on a standard natural fruit host, bananas (Kamala Jayanthi and Verghese, 2002) in the Entomology laboratory of Crop Protection Division at the ICAR-Indian Institute of Horticultural Research, Bangalore, India. Fifth-generation laboratory-cultured newly emerged adults were kept in an isolated room away from any odors in wooden nylon-net cages (30 cm × 30 cm × 30 cm). They were fed a diet of yeast extract powder (HIMEDIA, India) and sugar (1:1) separately along with water (provided on cotton swabs) *ad libitum*. The flies were allowed to mature and mate. The gravid females aged 15–20 days were used in the experiments.

### Oviposition Substrate

Agar plates (2%) were made aseptically in a laminar flow hood using gamma-irradiated sterile PHP plates (90 mm VWR, United States). Warm sterile agar made in distilled water (20 mL) was poured into the petri plates and allowed to solidify. These plates were used as oviposition substrates for all studies wherever applicable.

### Behavioral Studies

*Bactrocera dorsalis* females (*n* = 10) were collected and chilled on ice for 3–4 min until they were inactivated. The inactive insect was held gently with their wings using blunt forceps and decapitated (at the junction of head and thorax) using a pair of sterile and sharp microscissors. These insects were then placed in individual agar (2%) plates smeared with GOL. The activity of the decapitated insects was observed continuously, and visual observations were recorded on the sequence of various behaviors displayed. Behavioral activities of the decapitated insects were observed, and results obtained were tabulated as average frequencies for each observed event. The frequencies were converted to transition frequencies and compiled in a matrix to plot a Circos^1^ (Krzywinski et al., 2009), which depicts the transition of events in the decapitated insects. Observations were also done on the survival period (days) of decapitated female flies.

### Oviposition Bioassays

Four different sets of experiments were carried out, and each set was replicated ten times (*n* = 10). In the first set, gravid females were placed on ice in tubes for 3 min before transferring to the agar plate. In the second set, gravid females were taken, placed on ice as mentioned above, and then carefully decapitated using a pair of microscissors with minimal stress. The decapitated females were then placed on the agar plates. Only insects that were capable of standing erect on the plate were chosen for the experiment. In the third and fourth sets, intact and decapitated females were transferred to plates smeared with 10 μl of 1,000 ppm GOL, a known oviposition stimulant of *B. dorsalis* (Kamala Jayanthi et al., 2012, 2014a,b, 2017). In all experiments, flies (both intact and decapitated) were released into the agar plates in batches of five. All the agar plates were kept separated from each other in different rooms and allowed to sit for 24 h. The egg clusters were counted for each plate after 24 h. The data were tabulated and analyzed using one-way ANOVA and Tukey’s multiple comparison test (GraphPad Prism, v7.03). Survival was also recorded for all the flies until they lived.

### Electroantennogram and Electroovipositogram Studies

Electrophysiological studies to understand the antenna and also ovipositor response of female *B. dorsalis* to different dilutions of the known oviposition stimulant GOL (0.001 to 100 μg) were performed using electroantennogram (EAG) and electroovipositogram (EOG), respectively.

#### Preparation of Insect Antennae

The head of a gravid *B. dorsalis* female (*n* = 10), anesthetized by chilling, was separated from the body with a microscissors. EAG preparations were obtained by excising the pair of antennae from the head with a pair of microscissors and placing them on the EAG probe in a manner, which ensures that the antennal base is in contact with the indifferent ground electrode, and the other end touches the recording electrode. The prepared EAG probe was then inserted into the preamplifier with a constant stream of humidified air over the antenna at 200 mL min^–1^. The signals were passed through a high-impedance amplifier (Syntech, Germany, IDAC-4) and analyzed using a customized software package (Syntech).

#### Preparation of Insect Ovipositor

*Bactrocera dorsalis* females were collected in plastic vials by carefully trapping them between the vial opening and the nylon-net of the rearing cage and allowed to starve for 2 h. For ovipositor preparation, each individual female fly was held with a pair of forceps and a slight pressure was applied to the abdomen. The aculeus that protruded out was excised using microforceps and placed on the electrode. The ovipositor was cleaned of any sheath covering it and was placed on the EOG–EAG probe holder (Syntech, Germany), with a small amount of electrode gel (Signa Gel, United States), which ensures that the tip of the ovipositor was on the recording electrode and the base was on the indifferent ground electrode (Yadav and Borges, 2017). Thus, the prepared EOG probe that contains the excised ovipositor was then inserted into the preamplifier with a constant stream of humidified air over the ovipositor at 200 mL^–1^.

#### Odor Stimulus

γ-Octalactone (97% pure), a known oviposition stimulant for *B. dorsalis*, was procured from Sigma-Aldrich (India) and diluted in n-hexane (99.9%, Merck, India) to obtain different dilutions (100 to 0.001 μg) for EAG and EOG experiments.

#### Electroantennogram–Electroovipositogram Recordings

Electroantennogram and EOG recordings were performed as previously described (Cork et al., 1990; Kamala Jayanthi et al., 2021). To prepare the antennal–ovipositor odor stimuli, 10 μl of test sample (GOL) was pipetted out onto separate filter paper strips (Whatman No. 1, 6 cm length × 1 cm width) and allowed to evaporate for 1 min before placing the filter paper inside the glass Pasteur pipette (10 cm length and 6 mm outer diameter). Stimulation of the antennal–ovipositor preparation was carried out using controlled airflow (300 mL min^–1^) through the pipette with the filter paper. By injecting a puff of purified air (1 s), odor stimulation was administered, amplified, and recorded using the AutoSpike software of the Syntech EAG Model IDAC-4 (intelligent data acquisition controller). Purified air was passed over the antennal–ovipositor preparation for at least 30 s between stimulus presentations. The configuration in the AutoSpike properties tab for the channel with the EAG probe was set at a sampling rate of 100 and a filter of 0–32 Hz.

In this assay, air and honey were used as negative and positive controls, respectively (Subhash et al., 2018; Subramani et al., 2021). Normalization was done by stimulating with honey at the beginning and the end of each recording for the loss of sensitivity of the antennal preparation. Similarly, a control n-hexane stimulation was done at the beginning and at the end of each recording to subtract the blank value from the antennal responses (Ren et al., 2017). Antennal and ovipositor responses were recorded for different dilutions of GOL (0.001to 100 μg) based on the deflection signal (in mV) using the Syntech software. For each dilution, EAG response for 10 ovipositor–antennae of *B. dorsalis* was recorded. The data (signal means in mV) were subjected to non-parametric Friedman’s test (SPSS v 28.0), correlation and regression analyses (GraphPad Prism, v7.03).

### Ribonucleic Acid Isolation

Ribonucleic acid (RNA) extraction was carried out for different treatments, namely, the unexposed intact (U), unexposed decapitated (UD), GOL-exposed intact (E), and GOL-exposed decapitated (ED) using RNeasy Mini Kit (Qiagen) according to the manufacturer’s protocol with slight modifications (the insect samples were homogenized using micropestles in 1.5-mL microfuge tubes, and the RNA was eluted in 30 μl of nuclease-free water). RNA integrity was verified by 1.5% gel electrophoresis, whereas purity and concentration were determined using NanoDrop (DeNovix DS-11 spectrophotometer, Wilmington, DE, United States).

### Differential Expression Analysis of Oviposition-Related Genes Through qRT-PCR

Eight interesting oviposition-related genes (Table 1) based on insect literature [*Drosophila, B. dorsalis*, *Diaphorina citri* Kuwayama, *Locusta migratoria* (L.)] survey along with the reference gene, 18srDNA (GenBank accession no. AF033944.1), were selected for expression analysis. The sequences were obtained from NCBI database^2^. All qRTPCR primers that include the 18srDNA gene primers were designed using RealTimeDesign qPCR Assay Design Software^3^ and tested for PCR efficiency by melt curve analysis. The total RNA extracted was treated for genomic DNA contamination at two different stages: A. during extraction, since the kit includes a genomic DNA removal step with specialized DNAse columns. B. post-elution, as the cDNA synthesis kit (Qiagen Inverse strand kit Qiagen catalog no. 205310: United States) also has an additional genomic DNA removal step. The genomic DNA-free samples were used for downstream analysis. Synthesis of cDNA was performed with 1 μg total RNA using the Qiagen Inverse strand kit (Qiagen catalog no. 205310: United States) following the manufacturer’s protocol. Realtime analysis was performed on the Applied Biosystems Step One Plus machine. Two biological replicates with three technical replicates each were used for the expression analysis. Ct values obtained were exported to Excel, and the ddCt method was followed to compare the expression profile. 2^∧^-ddCt value was represented as a histogram against the target gene. A paired *t*-test was performed to estimate the *p*-values, and standard error (SE) was plotted as error bars on the histograms.

**TABLE 1:** Genes for realtime expression analysis.

**Gene of interest**	**Accession No.**	**Primer name**	**Primer sequence**
18 S rDNA	AF033944.1	BD18sqrtF	ACGACGCGAGAGGTGAAAT
		BD18sqrtR	GATCGCCTTCGAACCTCTAAC
*Octopamine Beta receptor 1* (*Oct*β*1R*)	GAKP01001054.1	BDOCBR1qrtF	GGACGATCAGCCGATGTATTTAGG
		BDOCBR1qrtR	CCACTGCCATTACCGTCTTCA
*Octopamine Beta receptor 2*(*Oct*β*2R*)	GAKP01016643.1	BDOCBR2qrtF	GACTGCGCTACCAACAACAG
		BDOCBR2qrtR	ACTGCCGCACGCATTGTC
*Octopamine Beta receptor 3*(*Oct*β*3R*)	GAKP01000600.1	BDOCBR3qrtF	GCCGCATCGCAAGAATTCAC
		BDOCBR3qrtR	GTCGCGTATTCGCTGATATTGC
*Proctolin* (*Proc*),	GDRP01022813.1	BDProctolin1qrtF	GCCATGCGAGGGACGTTA
		BDProctolin1qrtR	TCGCGCAATTTATCCAAATCGT
*Ribosomal proteinL13a*	GAKP01001216.1	BDRPl13AqrtF	GCCAAGTTGGACGTTTGTCA
		BDRPl13AqrtR	CCTTGCGTTTCCTTTCCAGACT
*Transformer-2* (*Tra2*)	GAKP01003494.1	BDTra2qrtF	GCTCCGGCATGGAAATTGATG
		BDTra2qrtR	GTCGGCCCATATAGACACCAG
*Tyramine beta hydroxylase* (*TBH*)	GEYS01020823.1	BDTBHqrtF	AGTCTTCTGGCTGCTGAAACTAT
		BDTBHqrtR	TTCCACAAGCGAGCTTAGAAA
*Octopamine/Tyramine receptor 1* (*Tyrr1*)	GDRP01007664.1	BDTyrr1qrtF	AACGCCGCCACAATCTTC
		BDTyrr1qrtR	ACGCCTGCTGGTTTCTTC
*Vitellogenin* (*Vit*)	GAKP01013338.1	BDVitlgnqrtF	ACACGCCTAACTGGTAGACAA
		BDVitlgnqrtR	CGTCGTTTGAATTTCGCCATAATCG

## Results

### Oviposition Assays

**FIGURE 1:**
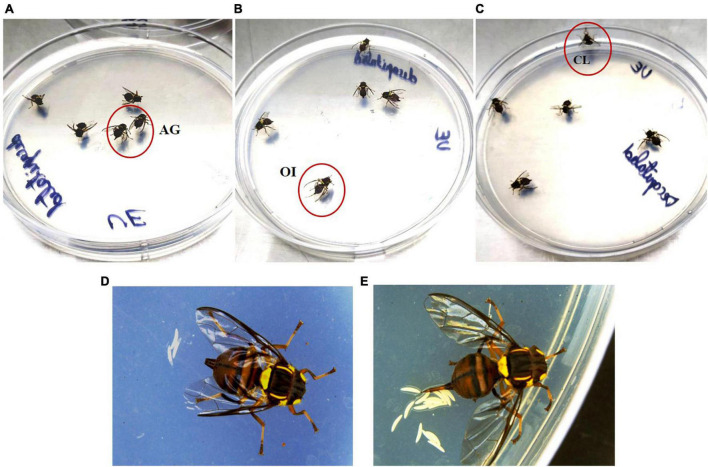
Decapitated female *B. dorsalis* can oviposit: **(A–C)** Decapitated female flies released on agar plate smeared with oviposition stimulant GOL. The flies in red circles are displaying some behavioral events such as abdomen grooming (AG), ovipositor insertion (OI), and climbing plate wall (CL) **(D,E).** The eggs laid by decapitated gravid female *B. dorsalis*.

**FIGURE 2:**
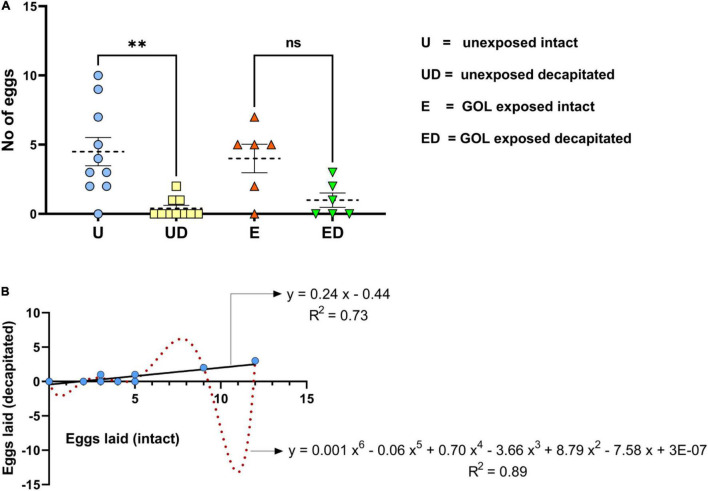
Ovipositional bioassays for *B. dorsalis*, **(A)** The number of eggs laid [*y*-axis] was plotted for *B. dorsalis* intact [blue circles]; decapitated [yellow squares]; intact and GOL exposed [orange triangles]; and decapitated and GOL exposed (green downward triangles). Gravid intact female flies laid significantly greater number of eggs (mean ± SEM; 4.50 ± 1.02; one-way ANOVA, Tukey’s multiple comparison test, *F* = 7.46, *df* = 3, ^∗∗^indicates significant difference with *p* = 0.001) compared to decapitated (0.40 ± 0.22), whereas no significant difference was observed between GOL-exposed intact (4.00 ± 1.03) and GOL-exposed decapitated (1.00 ± 0.51) *B. dorsalis* for the number of eggs laid. **(B)** The simple linear and polynomial (order 6) regression curves were plotted against the number of eggs laid by decapitated (*y*-axis) and intact (*x*-axis) female *B. dorsalis*. The trend line was constructed (black for linear and red for polynomial) with the regression equation and R-squared values depicted, respectively.

The main objective of the experiment was to test whether *B. dorsalis* are able to oviposit if decapitated. Interestingly, like intact females, decapitated females were also found to be active for a long time, and they were capable of laying eggs (Figure 1). Ovipositional bioassays carried out for a period of 24 h on intact and decapitated insects (unexposed and GOL exposed) revealed that whereas unexposed decapitated insects laid significantly fewer eggs (total 4 egg clusters, mean ± SEM; 0.40 ± 0.22) compared to the intact flies (total 45 egg clusters, mean ± SEM; 4.50 ± 1.02, *F* = 7.46, *df* = 3, *p* = 0.001), the decapitated females exposed to GOL were able to lay more eggs. When the decapitated flies were exposed to an oviposition stimulant (GOL), there were no significant differences between the treatments (total of 34 and 6 egg clusters with mean ± SEM; GOL-exposed intact = 4.00 ± 1.03; GOL-exposed decapitated = 1.00 ± 0.51, one-way ANOVA, Tukey’s multiple comparison test, *F* = 7.46, *df* = 3, *P* = 0.11) respectively (Figure 2A).

The correlation analysis of oviposition in intact and decapitated flies exhibited a significant positive correlation (*r* = 0.86, *p* = 0.01), which indicates a strong positive association. In other words, a shift in oviposition of GOL-exposed intact flies compared to unexposed fruit flies will likely be mirrored by the shift in oviposition of GOL-exposed decapitated flies compared to the unexposed. Thus, the egg-laying behavior of both intact and decapitated flies was quite alike before and after exposure to oviposition stimulant. The number of egg clusters was more in GOL-exposed intact flies compared to unexposed. Similarly, the number of egg clusters was more in GOL-exposed decapitated flies compared to unexposed. The regression analysis further corroborated this trend and revealed that maximum proportion of variability (*F* = 28.28, *df* = 10, *p* = 0.0003) in the egg-laying of decapitated flies (y) can be explained by the independent variable (here egg-laying of intact flies, x) with polynomial (*R*^2^ = 0.8931) and linear models (*R*^2^ = 0.7388) (Figure 2B). The quantitative variation in the egg-laying of decapitated flies (dependent variable) to the order of 89% (polynomial) and 74% (linear) can be directly attributed to variation in egg-laying of intact flies (independent variable), which suggests clear evidence of a trade-off between these two variables.

### Behavior of Decapitated Female *Bactrocera dorsalis*

**FIGURE 3:**
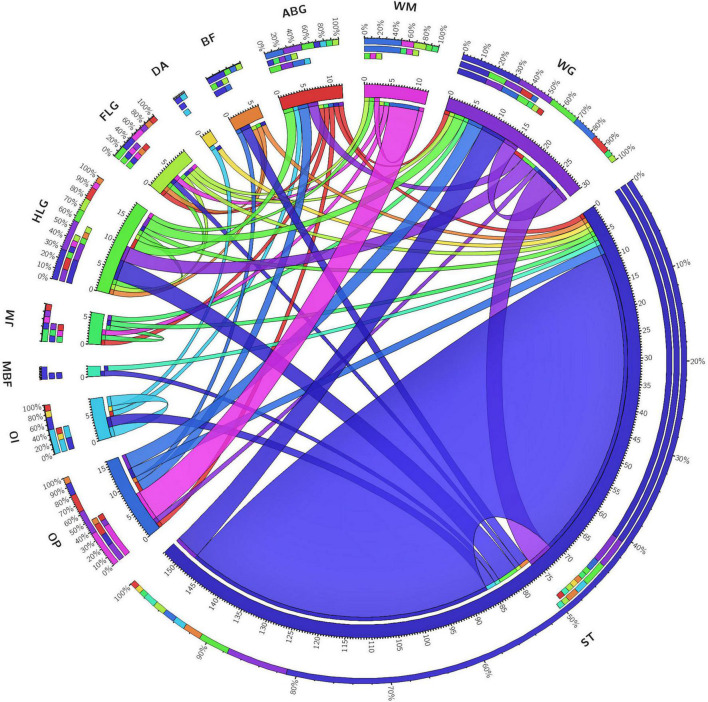
Behavioral transition events in decapitated *B. dorsalis* females: Behavioral events displayed by decapitated female insects were recorded as transition frequencies for the events, abdomen grooming (ABG), hind legs grooming (HLG), fore legs grooming (FLG), wing grooming (WG), bending in front (BF), random jerky movement (JM), ovipositor protruding (OP), ovipositor inserting (OI), dragging across (DA), moving back and forth (MBF), stationary (ST), and wing movement (WM). The matrix obtained was plotted as a Circos. The behavior pattern does not seem to follow any particular direction but shows events that would ultimately result in oviposition.

When decapitated, females were quite active and showed a variety of behaviors. Two-thirds of their time was spent in stationary phase (ST) and grooming of various body parts. Wing grooming (WG), grooming of the hind and fore legs (HLG and FLG, respectively), and abdomen grooming (ABG) were exhibited more frequently followed by ovipositor protrusion (OP) and ovipositor insertion (OI). General body movements, namely wing movement (WM), bending in front (BF), dragging across (DA), moving back and forth (MBF), and random jerky movement (JM), were also exhibited by decapitated females. The transition of events suggested that the insects displayed a behavioral pattern for oviposition. A majority of them also transitioned into a stationary phase for a longer time (Figure 3). The mean survival rate for intact females when not provided with food (set under similar conditions as decapitated females) was recorded at 3.80 ± 0.10 days (range: 5.00 to 8.00 days), whereas the mean survival rate for decapitated female *B. dorsalis* was recorded at 3.20 ± 0.26 days (range: 1.00 to 7.00 days), which was found to be statistically significant (*F* = 4.97; *p* = 0.04).

### Electroovipositogram and Electroantennogram

**FIGURE 4:**
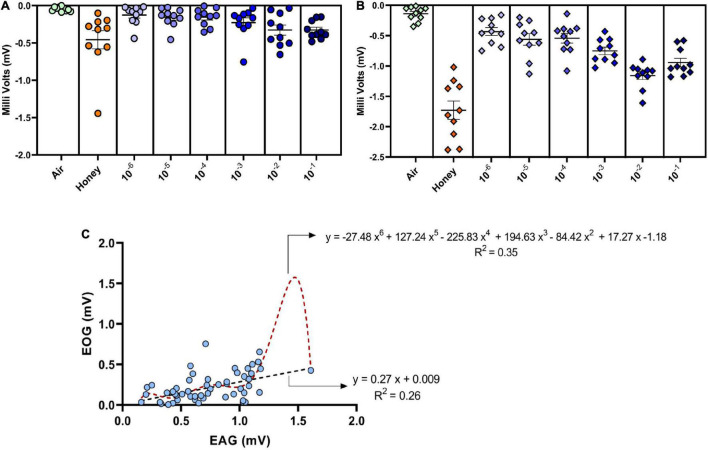
Dose-dependent electroovipositogram and electroantennogram responses of female *B. dorsalis*: **(A)** The EOG study showing the ovipositor response of *B. dorsalis* to different dilutions of GOL (10^–6^ to 10^–1^) analyzed through non-parametric Friedman’s test, χ^2^(7) = 29.26, *p* = 0.0001). **(B)** The EAG study for antennal response of *B. dorsalis* to different dilutions of GOL analyzed through non-parametric Friedman’s test, χ^2^(7) = 52.07, *p* < 0.0001). **(C)** Simple linear and polynomial (order 6) regression curves were plotted with the responses (in milli Volts) elicited by ovipositor (EOG, *y*-axis) against antennae (EAG, *x*-axis) of gravid female *B. dorsalis* for different dilutions of GOL. The trend line was constructed (black for linear and red for polynomial) with the regression equation and R-squared values depicted, respectively.

Response of the ovipositor to different dilutions of GOL was significant compared to the solvent. The 10^–2^ dilution evoked the strongest response (mean ± SEM; 0.99 ± 0.31; non-parametric Friedman’s test, χ^2^(7) = 29.26, *p* = 0.0001) in terms of the signal magnitude (Figure 4) similar to the trend observed in the antennal response (mean ± SEM; 1.15 ± 0.06; non-parametric Friedman’s test, χ^2^(7) = 52.07, *p* < 0.0001). Comparison of EAG and EOG responses revealed that the ovipositor and the antennal responses to GOL were in tandem, although the signals clearly showed a difference in the magnitude, which suggests that the antenna was more sensitive to the stimulant as expected.

The correlation analysis of EOG and EAG responses revealed a strong positive association (*r* = 0.52; *p* = 0.01) between them. Significant variability (*F* = 22.82, *df* = 62, *p* < 0.0001) in the ovipositor response can be explained [27 and 35 percent using the linear and polynomial (order 6) models, respectively] using antennal response as the independent variable. The observed trend indicates a strong defined relationship in the electrophysiological responses of both antenna and also ovipositor (Figure 4).

### Gene Expression

**FIGURE 5:**
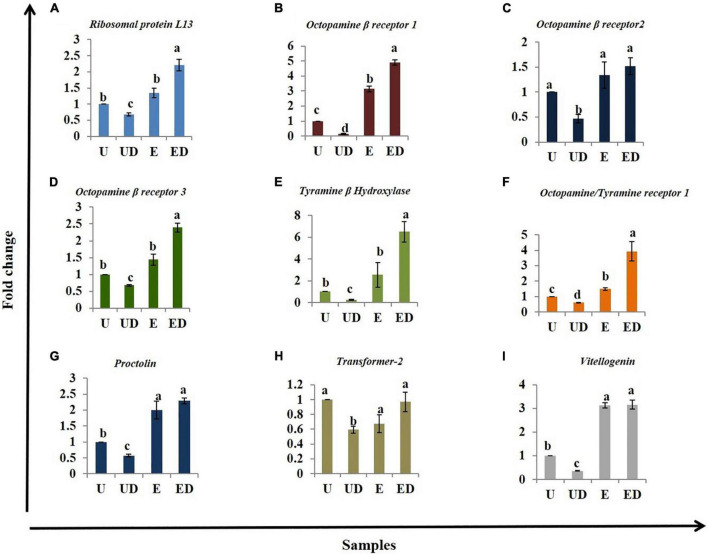
Realtime expression analysis of genes involved in oviposition: The different treatments, unexposed intact (U), unexposed decapitated (UD), GOL-exposed intact (E), and GOL-exposed decapitated (ED), were analyzed for different genes using qRTPCR analysis and the ddCt method. **(A)** The *Ribosomal proteinL13a* (*RPL13a*) is upregulated in GOL-exposed females (E and ED) and downregulated in UD. **(B)** *Octopamine beta receptor 1* (*Oct*β*1R*) is upregulated in GOL-exposed females (E and ED) and downregulated in UD, **(C)** *Octopamine beta receptor 2* (*Oct*β*2R*) is downregulated in UD, **(D)** *Octopamine beta receptor 3*(*Oct*β*3R*) is upregulated in GOL-exposed decapitated females (ED) and downregulated in UD, **(E)** *Tyramine beta hydroxylase* (*TBH*) is upregulated in GOL-exposed decapitated females (ED) and downregulated in UD, **(F)** *Octopamine–tyramine receptor 1* (*Tyrr1*) is upregulated in GOL-exposed females (E and ED) and downregulated in UD, **(G)** *Proctolin* (*Proc*) is upregulated in GOL-exposed decapitated females (ED) and downregulated in UD, **(H)** *Transformer-2* (*Tra2*) did not show any significant changes in expression across the treatments and **(I)** *Vitellogenin* (*Vit*) is upregulated in GOL-exposed decapitated females (ED) and downregulated in UD. Bars with similar alphabets are statistically non-significant.

A cascade of genes works in synchrony to make oviposition possible. Hence, based on the available literature, genes which are likely involved in oviposition were selected for expression analysis in the decapitated *B. dorsalis* females. *Ribosomal proteinL13a* (*RPL13a*), *octopamine beta receptor 1* (*Oct*β*1R*), *octopamine beta receptor 2*(*Oct*β*2R*), *octopamine beta receptor 3*(*Oct*β*3R*), *tyramine beta hydroxylase* (*TBH*), *octopamine-tyramine receptor 1* (*Tyrr1*), *proctolin* (*Proc*), *transformer-2* (*Tra2*), and *vitellogenin* (*Vit*) were profiled for expression analysis in the different treatments (U, UD, E, and ED) with 18srDNA as reference gene. Seven out of the nine genes were significantly upregulated in the GOL-exposed decapitated females (Figures 5A–I). Surprisingly, *RPL13a*, a stable housekeeping gene and a reliable reference gene (Collins et al., 2014; Shu et al., 2018), was found to be overexpressed in the GOL-exposed females (Figure 5A). The UD females showed significant downregulation of all genes compared to the U. Significant upregulation of *Oct*β*1R* (Figure 5B), *Tyrr1* (Figure 5E), *Proc* (Figure 5G), and *Vit* (Figure 5I) was observed in the GOL-exposed females compared to the intact unexposed females. The *Tra2* gene, which is responsible for sex determination in insects (Goralski et al., 1989; Yu and Killiny, 2018), did not show any significant change in the exposed samples compared to the unexposed (Figure 5H). It did show lowered expression in the UD compared to U. The results suggest that expression of some genes related to oviposition in other insects and likely also in *B. dorsalis* analyzed here is affected by decapitation and/or exposure to GOL.

## Discussion

Insects are fascinating with respect to their resilience (Middleton and Latty, 2016). A major advantage that works in their favor is the proficiency with which they can reproduce (Engel, 2015). Decapitation in insects does not lead to their death immediately because they have a relatively decentralized circulatory, nervous, and respiratory system (Litwack, 2012). Some pioneering work done several decades ago in *Mantis* (Roeder, 1935) and *Aedes* (McDaniel and Horsfall, 1957) provides evidence for facilitated mating in decapitated males whereas decapitation in female crane flies was found to initiate oviposition (Chiang and Kim, 1962). More recently, a precise kicking behavior was observed in intact and decapitated *Drosophila* in response to brushing of the recurved bristles on the wings by a mite (Li et al., 2016). Studies with *Anastrepha suspensa* and *Bactrocera tryoni* show that males copulate with decapitated females as well, and there is no significant difference in the sperm storage ability (Fritz, 2009; Pérez-Staples et al., 2010). Thus, the control of *copula* duration has been explored in decapitated tephritids but no studies have been done on oviposition as far as we know. Do the effects of decapitation on insect oviposition behavior suggest the occurrence of a bypass pathway? Curiosity to address this question drove us to begin exploring this aspect in the tephritid fruit fly, *B. dorsalis*, a notorious pest of several fruit crops (Clarke et al., 2005) and a prime focus of our laboratory. Using *B. dorsalis* as a model system, our research group is trying to understand the chemoecological behavior of this insect with respect to host attraction and oviposition (Kamala Jayanthi et al., 2012, 2014a,b, 2017, 2021). The work reported here was initiated to understand the contribution of brain in the oviposition behavior of *B dorsalis*.

In this study, the decapitated insects survived for long periods and exhibited normal behavioral events such as grooming legs, wings, abdomen, and ovipositor. Our data were able to record certain behaviors such as ovipositor grooming, hind leg grooming, and abdomen grooming, and these were captured in a video recording (Supplementary Material 1). Further, the use of an ovipositional stimulant like GOL revealed that the decapitated flies could perceive the presence of an ovipositional stimulant and lay more eggs compared to when there is no exposure to ovipositional stimulant, which highlights that oviposition could potentially have a bypass pathway and occur independently of the brain. Thus, exposure to GOL affected the egg-laying behavior in *B. dorsalis* females. This is an interesting result because in the absence of main organs such as antennae, these flies could still sense the ovipositional stimulant.

Insects carry innate memory of the ovipositional stimulant cues which are the part of the host volatiles that support their progeny survival (Gregório et al., 2012; Kamala Jayanthi et al., 2014a). This opens up an interesting debate that oviposition site selection in *B. dorsalis*, which is often mediated through innate recognition templates (IRTs) that are tuned to GOL (Kamala Jayanthi et al., 2014a), can also function independent of brain center. Thus, oviposition post-decapitation in *B. dorsalis* could be a molecular and coordinated maternal drive. This was also evident from the fact that the excised ovipositor could respond to GOL in the EOG experiments. SEM studies done previously on different appendages of tephritids including *B. dorsalis* (Zhang et al., 2012; Liu et al., 2021) reported differences in the type and density of sensillae on antennae and ovipositor. Significant difference in the density of the sensillae on the ovipositor compared to the antenna did not abolish the ovipositor response to GOL, which was comparable to that of the antenna. Further, the results of the EOG response to GOL indicate that even in the absence of antennae, there may be a fallback system to detect an oviposition site. This fallback system could potentially suffice to ensure that the oviposition site is suitable for the progeny.

Neural connections could be lost on decapitation, which possibly leads to loss of several functions in insects such as loss of tone (Roeder, 1937), spontaneous walking, neuronal activity (Schaefer and Ritzmann, 2001), and coordination of leg movements (Bässler et al., 1985; Ridgel and Ritzmann, 2005). Separation or detachment of the ventral nerve cord between thorax and abdomen results in loss of ability to oviposit in female crickets (Carrow et al., 1982). The role of specific neurons involved in oviposition and extrusion of eggs has been studied using targeted genetic tools in *Drosophila* (Kimura et al., 2015) but no such studies have been done in economically important tephritids nor are genetic tools available. Generally, in insects, the post-mating switch that leads to egg-laying involves a cascade of events such as rejection of mates, search for oviposition sites, assessment of threats, food sources for the progeny, etc. (Cury et al., 2019). The act of oviposition itself could involve the synchronous regulation of several molecular elicitors that lead to the final act of oviposition and egg extrusion. The role of some neurotransmitter pathway-related genes was explored in this study since work reported earlier on decapitated insects in terms of oviposition behavior seems to suggest that the insect nerve cord has controls that have been taken over by the forebrain in higher-order animals (Yellman et al., 1997). Therefore, understanding the molecular elicitors involved would be important to separate each functional aspect. Results of the expression profiling clearly indicate that the decapitated females exposed to the oviposition stimulant have misregulated expression of genes involved in oviposition. Biogenic amines have been shown to play an important role in conserved behavioral response in decapitated *Drosophila* (Yellman et al., 1997). Therefore, we chose to explore receptors for octopamine and tyramine. Among these, neurotransmission-related genes such as *TBH*, *octopamine beta receptors*, and the *octopamine–tyramine receptors* were upregulated in GOL-exposed decapitated females. The same was not true for the control and GOL-exposed intact females, which suggests that there is a possible switch in the distribution of tissue resources when the brain center is missing. The results indicate the existence of a bypass pathway for oviposition events that does not require the brain center and the nerve chord may suffice for its functionality. The *proctolin* gene that encodes for a neuropeptide also works as a co-transmitter associate coordinating ovulation and oviposition (Lange, 2002). The expression profile also revealed *RpL13a*, a ribosomal protein involved in translation repression in response to stress (Kapasi et al., 2007) to be upregulated in decapitated insects. Such conditions could trigger a series of reactions that lead to specific events as a salvage strategy (in this case oviposition). The oviposition bioassay data suggest that decapitated females do not lose the ability to oviposit, and a bypass nervous control other than the brain center works toward achieving this. The molecular data indicate a cascade of genes that are affected by decapitation and exposure to an oviposition stimulant which triggers extrusion of eggs. In its entirety, our data suggest that *B. dorsalis* retain the ability to oviposit even in the decapitated state since the nervous system and the circulatory system are relatively decentralized in insects. Results reported here for intact and decapitated *B. dorsalis* flies provide evidence that decapitation and succeeding oviposition in these insects might not be a simple reflexive action as reported earlier (M’Cracken, 1907; Spieth, 1966; Grossfield and Sakri, 1972) but a coordinated drive (reviewed by Lange, 2009). Usually, a reflexive action is immediate and instinctive. The egg-laying behavior displayed by the decapitated insects in this study was not instinctive–immediate but rather a coordinated event involving assessment of the egg-laying medium, grooming of its body parts including the ovipositor and response to the ovipositional stimulant. Hence, it took some time for the entire act. The molecular data from gene expression analysis also suggests that the elicitors coordinated the entire event and resulted in a drive for oviposition.

The characteristic behavior reported in this study could potentially be a unique one since recent reports on western corn rootworm suggest that these pests lose the ability to oviposit when decapitated (Spencer and Orellana, 2020). Some neuronal receptors profiled here did show differential expression in decapitated insects but exploring the entire neurotransmission pathways in detail with respect to oviposition would help to understand egg-laying behavior and also support the development of improved strategies for pest control in these agriculturally important insects.

## Conclusion

Our results show that gravid *B. dorsalis* females are capable of surviving and oviposition even in decapitated state, which indicates this act is brain-independent. The decapitated flies responded to an oviposition stimulant (GOL) even in the absence of brain and antennae highlighting that oviposition site selection in *B. dorsalis* at least partly may be controlled by different sets of neuronal system that does not rely on the brain. The molecular data revealed that a cascade of genes such as *TBH*, *octopamine beta receptors*, and the *octopamine–tyramine receptors* with homologs in *Drosophila* too could potentially play a role in the act of oviposition under decapitated state. The observed results could potentially be a consequence of the relatively decentralized nervous and circulatory system in insects. Advanced molecular experiments involving knockdown analysis of above-identified genes and the neuronal network they interact with could potentially provide insightful details about the bypass pathway for oviposition.

## Data Availability Statement

The original contributions presented in the study are included in the article/Supplementary Material, further inquiries can be directed to the corresponding author/s.

## Author Contributions

MV and PDKJ designed the project and edited the manuscript. MV and SP executed the work and drafted the manuscript. MV, PDKJ, and SP contributed to the analysis and interpretation of the data. PDKJ provided the funds, logistics, laboratory support for this study, and supported by the ICAR- National Fellow grant. All authors contributed to the article and approved the submitted version.

## Conflict of Interest

The authors declare that the research was conducted in the absence of any commercial or financial relationships that could be construed as a potential conflict of interest.

## Publisher’s Note

All claims expressed in this article are solely those of the authors and do not necessarily represent those of their affiliated organizations, or those of the publisher, the editors and the reviewers. Any product that may be evaluated in this article, or claim that may be made by its manufacturer, is not guaranteed or endorsed by the publisher.

## Footnotes

1
http://circos.ca/intro/tabular_visualization

2
https://www.ncbi.nlm.nih.gov/

3
https://www.biosearchtech.com/support/tools/design-software/realtimedesign-software
